# Sex-Specific Effects of Birth Weight on Longitudinal Behavioral Outcomes: A Mendelian Randomization Approach Using Polygenic Scores

**DOI:** 10.1016/j.bpsgos.2024.100387

**Published:** 2024-08-26

**Authors:** Lars Meinertz Byg, Carol Wang, John Attia, Andrew Whitehouse, Craig Pennell

**Affiliations:** a School of Medicine and Public Health, College of Health, Medicine and Wellbeing, University of Newcastle, Newcastle, New South Wales, Australia; b Mothers and Babies Research Program, Hunter Medical Research Institute, Newcastle, New South Wales, Australia; c Division of Medicine, John Hunter Hospital, Newcastle, New South Wales, Australia; d Telethon Kids Institute, Perth, University of Western Australia, Western Australia, Australia; e Maternal Fetal Medicine, Division of Maternity and Gynaecology, John Hunter Hospital, Newcastle, New South Wales, Australia

**Keywords:** Aggression, Birth weight, Child Behavior Checklist, Children and adolescents, Developmental programming, Gene-environment interactions

## Abstract

**Background:**

It is unclear whether sex differences in behavior arising from birth weight (BW) are genuine because of the cross-sectional nature and potential confounding in previous studies. We aimed to test whether sex differences associated with BW phenotype were reproducible using a Mendelian randomization approach, i.e., association between polygenic score (PGS) for BW and behavior outcomes across childhood and adolescence.

**Methods:**

Using data from the Raine Study, we had 1484 genotyped participants with a total of 6446 Child Behavior Checklist assessments from ages 5 to 17 years. We used BW-PGSs in linear mixed-effect models to predict parentally assessed attention, aggression, and social problems scales; we also derived estimates and significance for a sex-by-genotype interaction. We used a Bonferroni-corrected significance threshold and tested robustness of the results with teacher assessments of behavior and a second PGS.

**Results:**

We found a sex-by-genotype interaction with lower BW-PGSs associated with increased aggression in males compared with females. These findings were consistent across various analyses, including teacher assessments. Surprisingly, a lower BW-PGS showed protective effects in females, while a lower BW phenotype had detrimental effects in males with evidence of a genotype-phenotype mismatch increasing aggression problems in males only.

**Conclusions:**

This study underscores the genuine nature of behavioral sex differences arising from low BW and highlights the sex-dependent and diverging effects of environmental and genetic BW determinants.

## Plain Language Summary

Previous studies have suggested a link between low birth weight and poor behavioral outcomes in children and adolescents, but it is unclear if these effects differ between males and females. We demonstrated that genes influencing birth weight had different effects on aggressive behavior in males and females with effects throughout childhood and adolescence; furthermore, our study suggests that birth weight environment and birth weight genes have different effects on behavior. In males, a mismatch between the genetically determined birth weight and the measured birth weight resulted in more aggressive behavior.

Mental illness is believed to be caused by gene-environment interactions and often begins in early childhood (1). Birth weight (BW) is associated with psychopathology and multiple diagnostic categories (2, 3, 4), but this association may be confounded by factors affecting BW and mental health outcomes. Notably, Mendelian randomization uses the random allocation of genes during meiosis as a natural experiment that removes confounding; this approach has replicated the association between lower BW and mental illness (5). Childhood behavior has also been associated with BW in multiple settings (6, 7, 8, 9), but the effects of BW could vary between individuals (10). Translational studies on developmental programming support these conclusions and have highlighted sex differences in programmable behavior outcomes (11,12).

Sex differences in psychiatric epidemiology are well established (13), and sex may also modify the behavioral effects of BW (14, 15, 16, 17); i.e., despite females having generally lower BW, sex may independently affect behavior. The largest study to date found that low BW increased attention and aggression problems only in males with a nonsignificant signal for social problems at school age (14), which was partly supported by a longitudinal study from ages 9 to 17 years (17). In contrast, another study reported that females with lower BW were at increased risk of attention problems at preschool age (15). Intriguingly, both low BW and biological sex are implicated as determinants of behavioral phenotype trajectories (longitudinal outcomes) across childhood and adolescence (18,19). This raises the possibility that biological sex does not change the behavioral phenotypes induced by low BW per se but simply the age at which these effects are manifested. In addition, measured BW is a variable with a substantial risk of residual confounding (20). Combined with the inconsistencies in previous studies, causal inferences on lasting sex differences in behavioral phenotypes from lower BW and potential mechanisms for this are premature.

Both genes and environment during gestation affect BW, and sex differences from low BW may stem from sex-by-environment or sex-by-gene interactions (21). To the best of our knowledge, the only twin study that tested sex differences in behavioral outcomes from low BW found no moderation from sex but looked only at attention-deficit/hyperactivity disorder symptoms (22). A sibling control design in Sweden found similar effect sizes of low BW on diagnostic rates of psychiatric illnesses for concordant male and female sibling pairs (23). The contrast between the related and the nonrelated population studies cited above may be explained by confounding or the use of lifetime diagnoses versus continuous behavioral assessments. Alternatively, behavioral outcomes from low BW may only show sex differences in more genetically diverse settings. Taken together, the evidence suggests that repeated measures across childhood and adolescence are needed to distinguish whether genetic determinants of BW drive lasting behavioral sex differences seen in low BW.

Our study sought to investigate whether a Mendelian randomization approach using polygenic scores (PGSs) for BW revealed different effects on behavior in males and females across childhood and adolescence focusing on attention, aggression, and social problems; this approach should be free of traditional confounders and allow more robust inferences about causation.

## Methods and Materials

### Study Population

**Figure 1:**
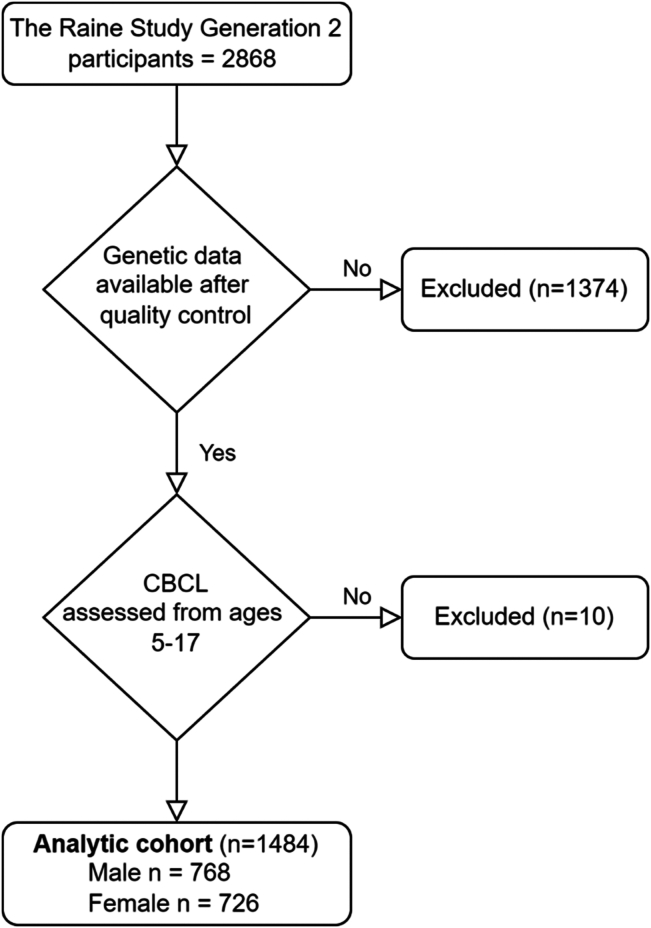
Flow diagram of cohort selection. CBCL, Child Behavior Checklist.

We analyzed data from the Raine Study (https://rainestudy.org.au/) (24). The Raine Study is a longitudinal study following mother-baby dyads recruited at or approximately 18 weeks’ gestation (*N* = 2979) through the public antenatal clinic at King Edward Memorial Hospital and nearby private clinics in Perth, Western Australia, from May 1989 to November 1991. Generation 2 (Gen2) participants were followed throughout childhood with modest attrition of mothers with lower age, education, income, and non-European ancestry (25,26). The Human Research Ethics Committees at the University of Western Australia, King Edward Memorial Hospital, and Princess Margaret Hospital in Perth, Australia, granted ethics approvals. Participants were eligible for analysis if they had data available on biological sex, at least one follow-up behavioral assessment, and genetic data (see Figure 1 for flowchart).

### Childhood Behavioral Assessments

Using parent reports of Child Behavior Checklist (CBCL) for Ages 4–18, we derived scores of attention (scale from 0 to 22), aggression (scale from 0 to 40), and social problems (scale from 0 to 16) at ages 5, 8, 10, 14, and 17 years. These 3 behavioral measures were chosen based on the report by Dooley *et al.* (14) who found sex differences in a cohort with similar ancestry for the majority population as ours (American vs. Australian). The CBCL for Ages 4–18 is a commonly used dimensional measure of child behavior during the previous 6 months based on parent ratings of 118 items on a 3-point Likert scale (0 = “not true,” 1 = “somewhat or sometimes true,” 2 = “very often or often true”) (27). For aggression problems, we also had available measures from the Achenbach System of Empirically Based Assessment preschool form from 2 to 3 years of age, included in sensitivity analysis. The 1991 edition of the preschool questionnaire did not have an attention problem and social problem scale, and the aggression score had more items (scale from 0 to 66) than the CBCL for Ages 4–18 (28). Assessments were excluded from analysis if they were missing more than 8 items on the entire CBCL (29), and missingness for an individual item was treated as a zero value. The attention problem subscale measures problems of attention, impulsivity, and hyperactivity and consists of 11 items. The social problems scale measures problems of peer interaction and consists of 8 items in total. The aggressive behavior scale measures both direct and relational aggression and consists of 20 items. The CBCL is a highly validated, clinically used psychometric tool, and the syndrome scales are reproducible across many countries (27,30, 31, 32). The teacher report form is another form by the same provider, used in the clinical setting to support findings from the CBCL (33). The teacher report form also has attention (20 items, score 0–40), aggression (25 items, score 0–50), and social problems (13 items, score 0–26) scales.

### Development of the BW-PGS

Information on Raine Study genotyping and quality control is provided in the Supplement. Genome-wide association analyses have enabled us to identify individual genetic variants associated with a wide range of traits; however, these effect sizes are usually small with low predictive power (34). Several studies demonstrated increased predictive power using PGS, a metric computed by summing the risk alleles associated with the phenotype of interest in each individual compared with a small number of genome-wide significant single nucleotide polymorphisms (35,36). In addition to identifying and understanding the potential etiology of disease, PGSs have also been used to test for genome-wide gene-gene and gene-environment interactions (35,37). To simplify the interpretation of the BW instrument, we used offspring genotype and identified 146 single nucleotide polymorphisms available in the Raine Study and associated with offspring BW from a 2019 genome-wide association study (GWAS) (38). These single nucleotide polymorphism data were extracted from the Raine Study Gen2 participants, recoded to correspond with increasing BW, weighted using the beta coefficients reported in the meta-analysis, summed, and rescaled before each participant’s BW-PGS was calculated. The final BW-PGS was then normalized (subtracting mean then dividing by standard deviation) to facilitate comparison with measured BW. For a sensitivity analysis, we generated a second BW-PGS based on variants identified from the BW GWAS published in 2016 (BW-PGS2) (39).

### Early-Life Determinants and Potential Confounders

Measured BW and fetal sex were retrieved from hospital records. For one participant with missing data, we extracted sex from a later questionnaire. The recorded BW was normalized for analysis to ease comparison with the BW-PGS. Preterm birth was defined as gestational age of <37 weeks, and gestational age was determined by either date of the last menstrual period or fetal biometry at the 18-week gestation ultrasound examination.

### Statistics

All statistical analyses and graph productions were performed in R and its associated libraries ggplot2, lme4, lmeresampler, and lmertest. Wilcoxon rank-sum test was used to compare sample distributions in the continuous baseline variables and CBCL outcomes between males and females.

To use the repeated behavioral measures most efficiently, we used linear mixed-effect modeling to test the associations between BW-PGS and behavioral outcomes; a random effect was used for participant ID and fixed effect for the BW-PGS and other covariables. Recent work has demonstrated that the treatment of ordinal data as continuous does not affect inference in most situations (40) including skew (41), and linear mixed modeling is robust to missing data and assumption violations (42). We treated the CBCL scores as continuous variables due to the dimensional nature of psychopathology (43). The clinical validation of the CBCL is always done with adjustment for age and sex (30), and therefore, the grouping variable for age at assessment was included in the regression giving us 2 models.

**Figure 2:**
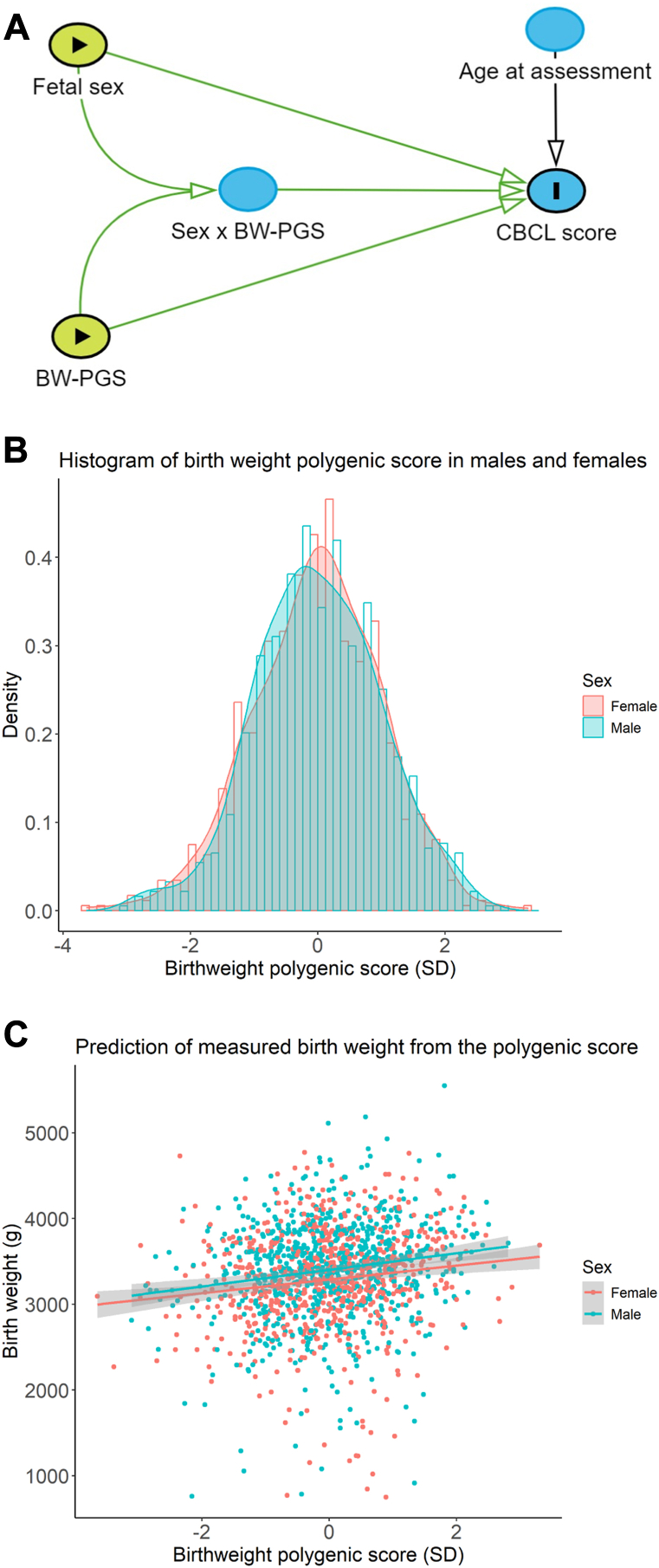
Directed acyclic graph and birth weight polygenic score (BW-PGS). Conceptual framework for the primary analysis demonstrated in a directed acyclic graph **(A)**. Density plot and histogram of the distribution of our BW-PGS in males and females **(B)** and the effect of the PGS on measured birth weight in males and females **(C)**. CBCL, Child Behavior Checklist.

Model 1 contains fixed effects of the BW-PGS and age at assessment, and model 2 contains the additional fixed effect for sex and a sex × PGS interaction (see Figure 2A for directed acyclic graph).

If $y_{IA}$ is the CBCL score for a participant ID at a specific age of assessment, $B_{0}$ is the grand mean intercept, $u_{I0}$ is our ID random effect on the intercept, $B_{1}$ is the coefficient estimate of ${PGS}_{IA}$ in females, $B_{2}$ is the interaction of male sex on $B_{1}$, ${\;B}_{3}$ is the fixed effect of age, $B_{4}$ is the fixed effect of sex, and finally, $\varepsilon_{IA}$ is the error term, then our regression equation for model 2 is as follows:

(1) $$y_{IA}\;=\;B_{0}\;+\;u_{I0}\;+\;B_{1}*{PGS}_{IA}\;+\;B_{2}*{PGS}_{IA}*{sex}_{IA}{+\;B}_{3}*{Age}_{IA}\;+\;B_{4}*{sex}_{IA}\;+\;\varepsilon_{IA}$$

We adjusted the models for the fixed effect of sex and PGS, while the PGS-by-sex interaction term in bold $B_{2}$ was the variable of interest for our primary research question. Due to our interest in 3 different behavioral outcomes, we applied a Bonferroni correction yielding a significance level of .01667 ($\alpha =\frac{0.05}{3}$) and the corresponding 98.3% CI was used for primary inference. To investigate the similarity in outcome-exposure relationships between our cohort and previous studies, we supplemented our analyses by running our models using the measured BW in the genotyped cohort as the exposure with adjustment for age at assessment and biological sex.

Model diagnostics were evaluated by histograms and quantile-quantile plots of residuals and random effects. After finding assumption violations in the quality control, a nonparametric bootstrap at the participant-ID level with 3000 simulations was used to derive standard error and 98.3% CIs as suggested by Tha *et al.* (44).

As sensitivity analyses, we reanalyzed our model with term-born offspring only and with the inclusion of an aggression assessment at age 2 years. Parents have clear associations with both the BW-PGS and CBCL, and we wanted to ensure the directionality of our findings with an independent analysis of teacher assessments at age 10 years. We also included a 3-way interaction with age to see whether the sex difference diminished with increasing age.

After finding large effect sizes for the BW-PGS, we reanalyzed our models using an earlier BW-PGS (BW-PGS2) (39) and added primary components to examine confounding from population stratification. After finding that nongenetic (i.e., environmental) factors account for the majority of variance in BW (21) and that BW-PGS has environment-sensitive effects (45), we sought to test whether there was an interaction between BW phenotype (reflecting primarily environmental determinants of BW) and BW genotype (reflecting genetic determinants of BW) in determining behavior. BW-PGS is causal for measured BW, and to avoid collinearity and bias in the interaction term, we generated a new variable “environmental BW” (BW-ENV and BW-ENV2 for BW-PGS and BW-PGS2, respectively) using the variance from a linear regression of measured BW on BW-PGS and BW-PGS2. We then tested for a genotype-phenotype interaction (BW-PGS × BW-ENV and BW-PGS2 × BW-ENV2) using a linear mixed model with inclusion of fixed effects for BW-PGS and BW-ENV for males and females separately and together.

## Results

The analytic cohort for the association between BW-PGS and behavior consisted of 1484 participants with outcomes for ages 5 to 17 years, corresponding to 52% of the original Gen2 Raine Study participants (Figure 1). We had 6446 CBCL assessments over the follow-up period. The average number of assessments was 4.35. A total of 874 participants had 5, and 25 participants just 1 assessment (Table S1). The maternal baseline characteristics were similar between male and female fetuses (Table S2). The genotyped cohort displayed selective attrition on a range of maternal baseline characteristics compared with the original Raine Study cohort, including lower BW (Table S3).

In our analytic cohort, males had 112-g higher BW than females and higher CBCL scores on the subscales of interest. In the genotyped cohort, males and females had a similar BW-PGS (*p* = .8), and the BW-PGS was normally distributed (Table S1 and Figure 2B). Without stratification for sex, a 1-SD increase in PGS resulted in an 88.9-g increase in BW (*p* < .0001), explaining 2.25% of the BW variance. This relationship had no significant sex interaction (*p* = .54) (Figure 2C). Analysis of the behavioral outcomes using measured BW (*n* = 1483) suggested a sex interaction where males had more behavioral problems at lower BW than females, but this interaction did not reach statistical significance (all interaction *p* > .0167) (Table S4).

**Figure 3:**
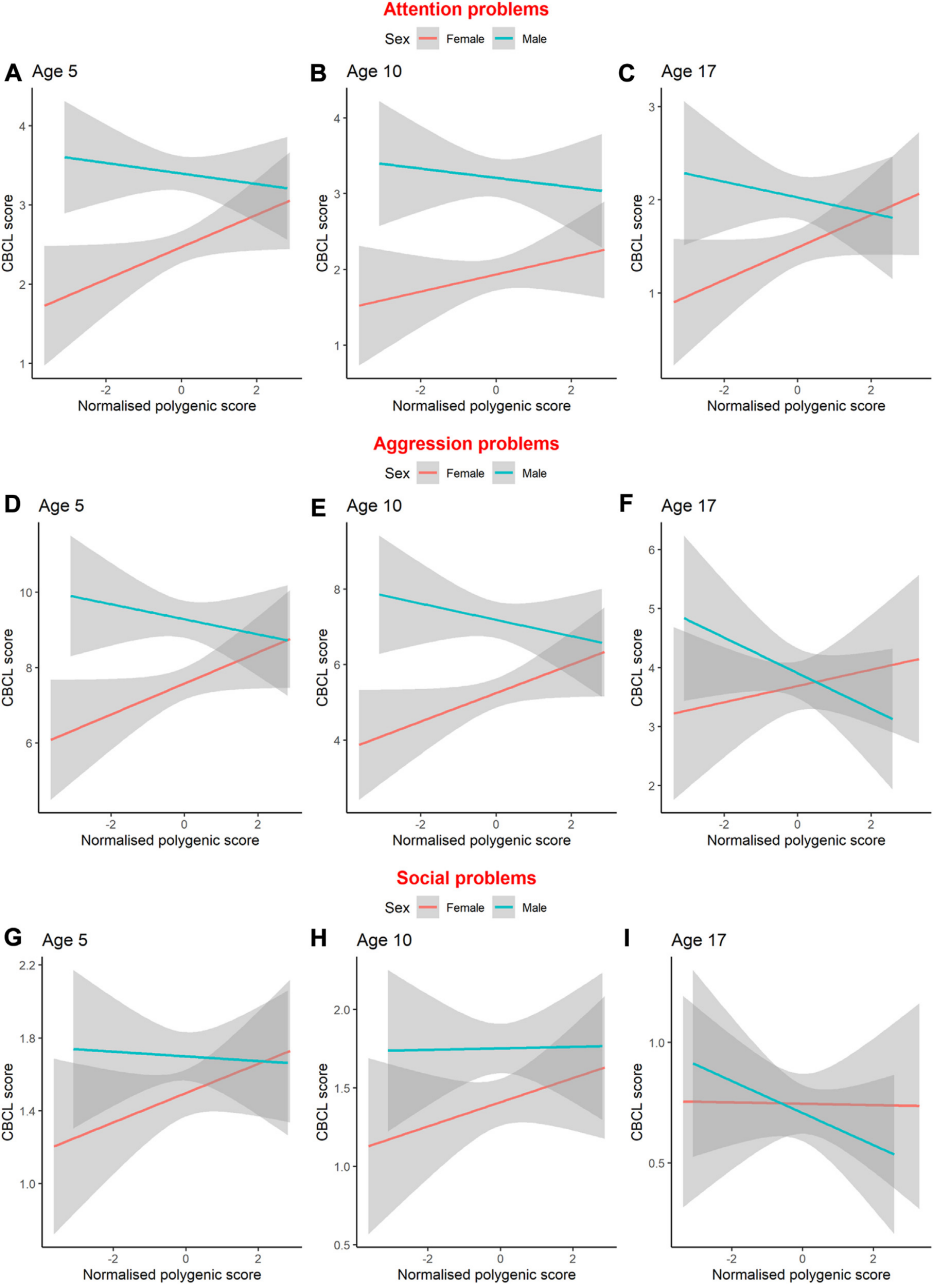
Graphical illustration of the sex interaction on behavioral effects of birth weight genetics. The linear relationship between the normalized polygenic score and attention **(A–C)**, aggression **(D–F)**, and social problems **(G–I)** is shown for ages 5, 10, and 17 years in males and females. Note that the y-axis changes depending on the Child Behavior Checklist (CBCL) syndrome and the age at assessment.

We found no main effect of the BW-PGS on aggression problems, attention problems, or social problems (*p* value for all >.0167) (Table 1). We then moved on to the primary research question regarding sex differences in the longitudinal behavioral effects of the BW-PGS. For aggression problems in females, a 1-SD increase in BW-PGS resulted in a 0.41-point increase (98.3% CI, 0.069 to 0.744; *p* = .0038) in CBCL aggression problems score, whereas in males a 1-SD increase in BW-PGS did not significantly affect aggression problems (B = −0.171; 98.3% CI, −0.534 to 0.188; *p* = .259) (Figure 3). The difference between female and male effects was significant given the formal interaction term was significant (*p* = .0045). For attention problems in females, a 1-SD increase in the BW-PGS resulted in a 0.179-point increase (98.3% CI, 0.019 to 0.331; *p* = .0063) in CBCL attention problems score, whereas in males, a 1-SD decrease in BW did not significantly reduce the effect of BW-PGS on attention problems (B = −0.00907; 98.3% CI, −0.184 to 0.158; *p* = .899). This difference between females and males was not significant at our Bonferroni-corrected threshold (*p* = .0498). For social problems, we found no significant effect of BW-PGS in females (B = 0.077; 98.3% CI, −0.0488 to 0.198; *p* = .138) or males (B = 0.033; 98.3% CI, −0.068 to 0.130; *p* = .421), and the interaction term was not significant (*p* = .52).

**Table 1:** Primary Analysis Output of Interaction Between Sex and Birth Weight Genotype

	Aggression Problems		Attention Problems		Social Problems	
	Model 1	Model 2	Model 1	Model 2	Model 1	Model 2
Combined Effect (BW-PGS)	B = 0.117 CI = −0.139 to 0.363 SE = 0.105, *p* = .266	NA	B = 0.085 CI = −0.034 to 0.200 SE = 0.05, *p* = .083	NA	B = 0.055 CI = −0.026 to 0.130 SE = 0.033, *p* = .091	NA
Female Effect (BW-PGS)	NA	B = 0.410 CI = 0.069 to 0.744 SE = 0.141, *p* = .0038	NA	B = 0.179 CI = 0.019 to 0.331 SE = 0.065, *p* = .0063	NA	B = 0.077 CI = −0.049 to 0.198 SE = 0.052, *p* = .138
Male Effect (BW-PGS)	NA	B = −0.171 CI = −0.534 to 0.188 SE = 0.151, *p* = .259	NA	B = −0.00907 CI = −0.184 to 0.158 SE = 0.0717, *p* = .899	NA	B = 0.033 CI = −0.068 to 0.130 SE = 0.0416, *p* = .421
Interaction From Male Sex on Female BW-PGS Effect	NA	B = −0.581 CI = −1.06 to −0.089 SE = 0.204, *p* = .0045	NA	B = −0.188 CI = −0.416 to 0.041 SE = 0.096, *p* = .0498	NA	B = −0.043 CI = −0.203 to 0.117 SE = 0.067, *p* = .52

Primary analysis showing the regression output for our linear mixed-effect model. *p* Value was calculated based on the *z*-statistic from the bootstrapped standard error. CI represents 98.3% CI.

BW-PGS, birth weight polygenic score; NA, not available.

As a prespecified sensitivity analysis, we included the preschool aggression problems assessment, yielding an additional 5 participants for analysis (*n* = 1489). Inclusion of this assessment was consistent with the primary analysis (Table S5). We then reanalyzed our model with the inclusion of term-born only (*n* = 1343) and saw only minor changes in effect estimates compared with the primary analysis (Table S6). We then wanted to confirm our results with teacher assessments at age 10 years (*n* = 1298). The sex × BW-PGS interaction was consistent with results from the primary analysis, suggesting that a decrease in BW-PGS resulted in increased CBCL scores for males compared with females (Table S7).

As an additional sensitivity analysis, we reanalyzed our models using a BW-PGS developed based on variants identified from the BW GWAS published in 2016 (BW-PGS2) (39). The BW-PGS explained 36% of the variance in the BW-PGS2, and the BW-PGS2 had a modestly better predictive value for measured BW (100 g per 1-SD increase) (Figure S1). We saw directionally similar results compared with the BW-PGS results, with effect estimates that were modestly reduced (Table S8). Adding principal components did not influence effect estimates (Table S9). Increasing age did not significantly diminish the sex interaction (Table S10).

**Figure 4:**
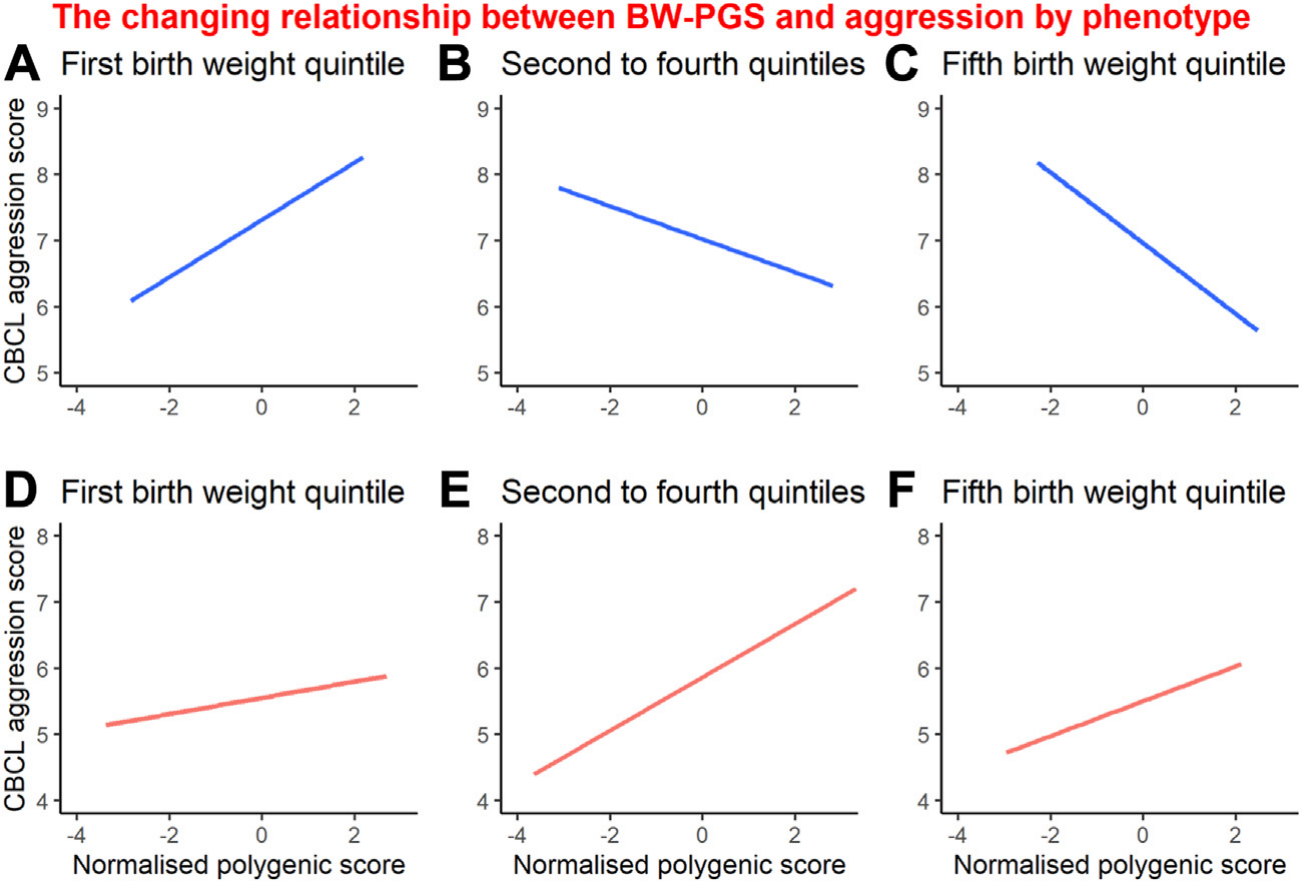
Birth weight (BW) phenotype-genotype interactions for aggression. In phenotypically smaller males (first BW quintile), a higher BW genotype increases Child Behavior Checklist (CBCL) aggression problem raw scores, whereas in phenotypically larger males (fifth BW quintile), a lower BW genotype increases aggression scores. This changing effect was statistically significant in males **(A–C)** but not in females **(D–F)**. Because of repeated measures, no confidence interval is given. PGS, polygenic score.

Finally, we investigated whether BW phenotype-genotype interactions affected behavior. BW-PGS and BW-ENV (measured BW adjusted for BW-PGS) had significant interactions when modeling aggression problems in males (Table 2), suggesting that higher PGS in the presence of lower BW-ENV resulted in increased scores of aggression compared with higher PGS in the presence of higher BW-ENV (Figure 4). This result was consistent when using BW-PGS2. We did not find similar interactions for attention or social problems (*p* > .05) (data not shown).

**Table 2:** Birth Weight Phenotype-Genotype Interaction for Aggression

	BW-ENV	BW-PGS	Interaction BW-PGS × BW-ENV	Interaction BW-PGS2 × BW-ENV2
Males and Females Combined	B = −0.148 CI = −0.410 to 0.117 SE = 0.11030971, *p* = .18	B = 0.117 CI = −0.132 to 0.359 SE = 0.10270252, *p* = .25	B = −0.146 CI = −0.396 to 0.102 SE = 0.10445926, *p* = .16	B = −0.315 CI = −0.550 to −0.0771 SE = 0.09912420, *p* = .001
Females Only	B = −0.115 CI = −0.429 to 0.207 SE = 0.1333038, *p* = .39	B = 0.418 CI = 0.0753 to 0.752 SE = 0.1417495, *p* = .003	B = 0.228 CI = −0.0977 to 0.548 SE = 0.1352507, *p* = .09	B = 0.0318 CI = −0.333 to 0.394 SE = 0.1523636, *p* = .83
Males Only	B = −0.194 CI = −0.591 to 0.196 SE = 0.1649299, *p* = .23	B = −0.133 CI = −0.498 to 0.227 SE = 0.1520547, *p* = .38	B = −0.416 CI = −0.781 to −0.0464 SE = 0.1538285, *p* = .007	B = −0.583 CI = −0.923 to −0.245 SE = 0.1421043, *p* = .00004

The interaction between measured BW and BW-PGS in a linear mixed-effect model. Models were made unstratified for sex and with males and females separately. *p* Value was calculated based on the *z*-statistic from the bootstrapped standard error. CI represents 98.3% CI. BW-PGS is the primary birth weight PGS used for analysis; BW-PGS2 is the secondary birth weight PGS made from an older GWAS; BW-ENV is the measured BW adjusted for BW-PGS; BW-ENV2 is the measured BW adjusted for BW-PGS2.

BW, body weight; ENV, environmental; GWAS, genome-wide association study; PGS, polygenic score.

## Discussion

Using repeated behavioral assessments across childhood and adolescence, we demonstrated 2 different interactions with BW: 1) a sex-by-genotype interaction showed increased aggression problems in males compared with females with lower BW genotype, and 2) a genotype-by-phenotype interaction showed differential effects on aggression depending on the discrepancy between genetically determined and environmentally achieved BW, suggesting that it is this mismatch that drives poor outcomes.

The current study is consistent with the established epidemiology of behavioral sex differences with higher CBCL scores in males for the behaviors of interest (30). The sex-by-phenotype interactions are consistent with results from most previous studies and suggest a male vulnerability at lower BW phenotype (14,16).

We found no evidence for a sex-by-genotype interaction in the development of social problems drawing the causal nature of this association into question. For attention problems, a nonsignificant signal suggested a male vulnerability at lower BW genotype, but this was inconclusive. This is surprising given the reproducible association between BW phenotype and attention-deficit/hyperactivity disorder (46) along with the sex interactions for the BW phenotype reported here and elsewhere (14,16). Our null finding could reflect insufficient power to detect the difference. Alternatively, it suggests that genetic determinants of BW are not important for the association between BW phenotype and attention-deficit/hyperactivity disorder, as proposed elsewhere (46).

The a priori hypothesis of a sex-by-genotype interaction causing increased CBCL scores at lower ranges of BW-PGS for males compared with females was confirmed when modeling aggression problems; however, the effect size of the sex interaction was unexpectedly large. A 1-SD increase in BW-PGS corresponded to 88.9-g change in measured BW (which had a 1 SD of approximately 580 g). If measured BW mediates the sex difference resulting from BW-PGS, this would mean that an unconfounded 1-SD decrease of measured BW would cause a 3.8-point higher score in aggression problems in males than in females. In the ABCD (Adolescent Brain Cognitive Development) cohort, a 1-kg change in measured BW caused a 0.38-point change in aggression problem scores for males compared with females—more than a 10-fold smaller difference than what we saw in our study. We saw sex interactions of similar magnitude in the teacher sensitivity analysis at age 10 years. Because of this large effect size, we created a second genetic instrument from an older GWAS, the BW-PGS2, which yielded results consistent with primary analysis.

When examining males and females separately, another surprising finding emerged. Although sex differences associated with BW phenotype resulted from a detrimental effect in males at the lower range, the sex difference seen in aggression problems was driven by a protective effect in females at the lower BW range. This protective female effect was also present for attention problems. This change in what drove the interaction (from males doing worse at lower BW to females doing better at lower BW) made straightforward mediation analysis of the BW genotype through BW phenotype invalid (no exposure-outcome or mediator-outcome association, respectively), suggesting that different causal pathways are at play. Environmental determinants of measured BW, such as smoking, have different effects on BW phenotype in males and females; therefore, this discrepancy could simply reflect confounding (47). Alternatively, we propose that a failure to meet one’s fetal growth potential has a detrimental effect in both males and females; however, at the population level, the consequence for females is masked by a protective effect from a lower BW genotype. This would also explain the discrepant results for genetically diverse populations versus populations with more genetically similar controls (23). The protective effect of BW genotype in females and the detrimental effect of BW phenotype in males is in stark contrast with Murray *et al.* (15) who reported a sex-by-phenotype interaction with a female vulnerability to attention problems at lower BW. Possible explanations for this discrepancy are the age 4 CBCL assessment, less favorable sociodemographic characteristics, and a lower range of phenotypic BW in the Pelotas birth cohort (48). Alternatively, measured BW could interact with the diverging genetics of ancestrally diverse populations (Brazilian and Australian) to change associations between BW phenotype and attention problems as reported by Rahman *et al.* (10).

We point out that our genotype-by-phenotype interaction was only detected post hoc, unlike our sex-by-genotype interaction, which was postulated a priori. Again, aggression problems emerged as a trait vulnerable to a genotype-phenotype mismatch with effects isolated to the males. Our results suggest that males with low BW phenotype are at risk of increased aggression problems only if they have a high BW genotype. This finding is supported by experimental growth restriction in primates leading to a male increase in aggression problems (12). The reversal of effect estimates from BW-ENV contingent on genotype question the ability of monozygotic twin studies to uncover the full range of developmental programming given that potential programming from BW discordance will depend on the shared genotype; however, this result should be seen in the light of 2 limitations. First, the variance of BW explained by genetics is estimated at 17% (21) while our genetic scores explained 2% to 3%; therefore, our results could represent a gene-by-gene interaction. The consistent effect when using the BW-PGS2 makes a gene-by-gene interaction more unlikely. Second, the BW environment variable was generated from measured BW, which is a confounded variable; however, the association was present in males only, so to bias our results, the underlying confounder architecture would have to differ by the random allocation of both sex and BW-PGS. Finally, our finding of a “frail male” is consistent with animal and human experiments and has been proposed to have a placental origin, as male and female placental responses to stressors are different (49, 50, 51).

The neurobiological mechanisms underlying sex differences in aggression related to BW are not elucidated by this study or, to our knowledge, any study; however, fetal neurodevelopment differs by offspring sex (52, 53, 54) and is in turn affected by BW (55). A study from the UK Biobank suggested that BW-PGS and measured BW affected adult depression through various brain regions and that these effects differed by offspring sex (56). Similarly, a recent study found that caudate nucleus volumes in neonates had opposite associations to PGSs for adult depression, providing evidence for sex-dimorphic relationships between fetal development and mental health (57). For aggression, a study of 193 children and adolescents suggested that the orbitofrontal and right anterior cingulate cortex volume had gender-specific associations to aggressive behavior. The morphology of the anterior cingulate and orbitofrontal cortex was associated with low BW (55) and extreme low BW (58). In light of our results, behavioral sex differences stemming from these effects warrant investigation.

This study has several strengths. Regressions using BW phenotype agreed with previous authors, suggesting that our exposure-outcome associations are generalizable. As accepted with Mendelian randomization designs, the BW-PGS should not be subject to confounding. The 3 behavioral outcomes were based on previous studies and tested at a conservative significance threshold. Prospective repeated assessments with a single validated instrument should increase accuracy and eliminate recall bias, and the teacher assessments provide evidence that our results are not caused by bias in parental assessments. The use of a second PGS to confirm our results supports the validity of our inference, and we saw robust results throughout the sensitivity analyses. Finally, we had a 99.3% follow-up of the genotyped participants.

Our limitations are the primarily Caucasian ethnicity in our cohort and the age 17 cutoff for our behavioral assessment; in addition, we saw normal distributions of the genetic scores, but the cohort without genetic information had different maternal baseline characteristics, which could reduce generalizability.

In conclusion, we have used robust causal inference methods to confirm that BW affects behavior in males and females differently across childhood and adolescence, and our results encourage incorporation of genetic scores in future twin studies. Our results also suggest that the discrepancy between genetically determined and environmentally achieved BW, i.e., mismatch, could be the driver of pathology rather than absolute BW or BW-PGS per se, but this will need to be confirmed in further studies. Finally, basic research into mechanisms underlying sex differences and gene-environment interactions in behavioral outcomes from environmental and genetic determinants of BW are needed to facilitate possible interventions.

## Supplementary Material

Key Resources Table
