# Supplementary material for: Sex-Specific Effects of Birth Weight on Longitudinal Behavioral Outcomes: A Mendelian Randomization Approach Using Polygenic Scores
Source: Biol Psychiatry Glob Open Sci. 2024 Aug 26;4(6):100387. doi: 10.1016/j.bpsgos.2024.100387 (PMC11526082; doi:10.1016/j.bpsgos.2024.100387)
Supplement: Supplementary Figure 1 and Supplementary Tables S1-S10 [file mmc2.pdf]

SUPPLEMENTARY INFORMATION

Sex-Specific Effects of Birth Weight on Longitudinal Behavioural Outcomes: A Mendelian Randomization Approach Using Polygenic Scores

Byg *et al.*

Contents

Supplementary Figure 1. Effect of the second polygenic score BW-PGS2 2
Supplementary Table 1. Behaviour- and birth weight variables 3
Supplementary Table 2. Baseline demographics males vs females 4
Supplementary Table 3. Analytic vs excluded cohort 5
Supplementary Table 4. Measured birthweight 6
Supplementary Table 5. Aggression with age two assessment 7
Supplementary Table 6. Term born only 8
Supplementary Table 7. Teacher assessments 9
Supplementary Table 8. Sex interactions when using BW-PGS2 10
Supplementary Table 9. Including principal components 11
Supplementary Table 10. Temporal stability of sex differences 12

Supplementary Figure 1. Effect of the second polygenic score BW-PGS2

a

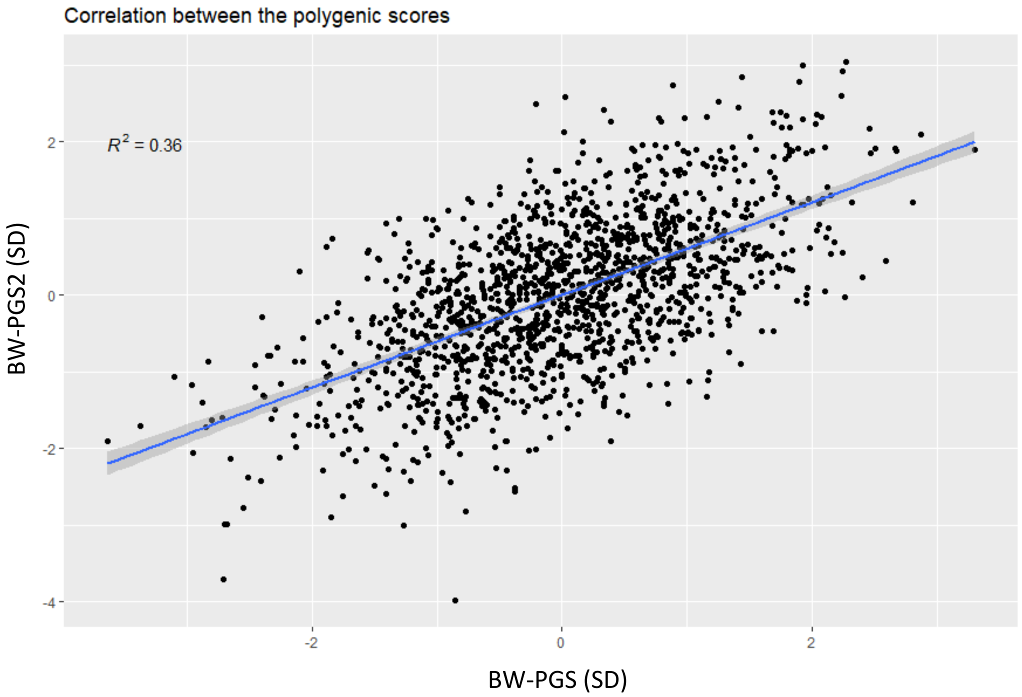

b

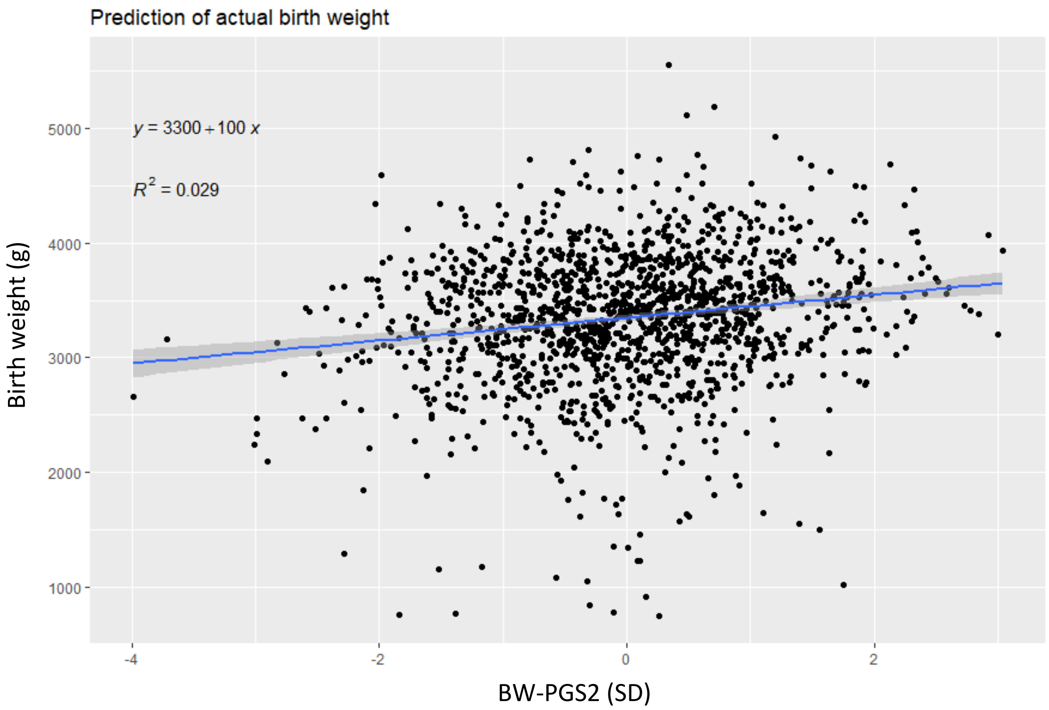

Supplementary Figure 1: The correlation between the BW-PGS used in primary analysis, and the second BW-PGS (BW-PGS2) used in sensitivity analysis (**a**) and the predictive value of the BW-PGS (**b**) for measured birth weight in our cohort in males and females combined. **BW-PGS**: Birth weight polygenic score.

Supplementary Table 1. Behaviour- and birth weight variables

Baseline information in Raine Study participants with genetic information and 1 or more Child behaviour checklist scores from ages 5 – 17 (n = 1484)

	**Female** (n = 722)	**Male** (n = 762)	**P-value***
**Measured birth weight (g)**			< 0.0001
Mean (SD)	3291.9 (±574.7)	3404.2 (±588.7)	
Missing	0 (0%)	1 (0.1%)	
**Polygenic score for birth weight (normalised)**			0.84
	0.0 (±1.0)	0.0 (±1.0)	
**CBCL Aggression problems age 5**			< 0.0001
Mean (SD)	7.6 (±5.6)	9.3 (±6.6)	
Missing	59 (8.2%)	62 (8.1%)	
**CBCL Aggression problems age 8**			< 0.0001
Mean (SD)	6.4 (±5.7)	8.3 (±7.0)	
Missing	65 (9.0%)	72 (9.4%)	
**CBCL Aggression problems age 10**			< 0.0001
Mean (SD)	5.2 (±5.1)	7.2 (±6.5)	
Missing	65 (9.0%)	53 (7.0%)	
**CBCL Aggression problems age 14**			0.043
Mean (SD)	5.3 (±5.8)	5.8 (±6.1)	
Missing	74 (10.2%)	79 (10.4%)	
**CBCL Aggression problems age 17**			0.6
Mean (SD)	3.7 (±4.7)	3.9 (±5.0)	
Missing	220 (30.5%)	225 (29.5%)	
**CBCL Attention problems age 5**			< 0.0001
Mean (SD)	2.5 (±2.6)	3.4 (±2.9)	
Missing	59 (8.2%)	62 (8.1%)	
**CBCL Attention problems age 8**			< 0.0001
Mean (SD)	2.4 (±2.8)	3.6 (±3.4)	
Missing	65 (9.0%)	72 (9.4%)	
**CBCL Attention problems age 10**			< 0.0001
Mean (SD)	1.9 (±2.7)	3.2 (±3.4)	
Missing	65 (9.0%)	53 (7.0%)	
**CBCL Attention problems age 14**			< 0.0001
Mean (SD)	1.9 (±2.5)	2.7 (±3.1)	
Missing	74 (10.2%)	79 (10.4%)	
**CBCL Attention problems age 17**			0.001
Mean (SD)	1.5 (±2.2)	2.0 (±2.7)	
Missing	220 (30.5%)	225 (29.5%)	
**CBCL Social problems age 5**			0.026
Mean (SD)	1.5 (±1.7)	1.7 (±1.8)	
Missing	59 (8.2%)	62 (8.1%)	
**CBCL Social problems age 8**			0.3
Mean (SD)	1.5 (±1.9)	1.7 (±2.1)	
Missing	65 (9.0%)	72 (9.4%)	
**CBCL Social problems age 10**			0.001
Mean (SD)	1.4 (±2.0)	1.8 (±2.1)	
Missing	65 (9.0%)	53 (7.0%)	
**CBCL Social problems age 14**			0.12
Mean (SD)	1.1 (±1.8)	1.3 (±1.9)	
Missing	74 (10.2%)	79 (10.4%)	
**CBCL Social problems age 17**			0.6
Mean (SD)	0.7 (±1.4)	0.7 (±1.4)	
Missing	220 (30.5%)	225 (29.5%)	

* P-value calculated using Wilcoxon-signed rank test

Supplementary Table 2. Baseline demographics males vs females

Demographics for Raine Study participants with genetic data and at least one CBCL score from age 5 - 17 (n = 1484)

	**Female** (n = 722)	**Male** (n = 762)
**Maternal age at birth (years)**		
Mean (SD)	28.8 (±5.9)	28.9 (±5.7)
Missing	11 (1.5%)	4 (0.5%)
**Income level†**		
Below 12.000 AUD	89 (12.3%)	90 (11.8%)
Above 12.000 AUD	589 (81.6%)	641 (84.1%)
Missing	44 (6.1%)	31 (4.1%)
**Maternal body mass index (kg/m^2)**		
Mean (SD)	22.5 (±4.3)	22.5 (±4.3)
Missing	11 (1.5%)	4 (0.5%)
**Maternal race**		
European descent	687 (95.2%)	737 (96.7%)
Other	24 (3.3%)	21 (2.8%)
Missing	11 (1.5%)	4 (0.5%)
**Maternal level of education**		
< 12 years	350 (48.5%)	351 (46.1%)
> 12 years	361 (50.0%)	407 (53.4%)
Missing	11 (1.5%)	4 (0.5%)
**Diabetes or hypertension in pregnancy**		
absent	585 (81.0%)	620 (81.4%)
present	126 (17.5%)	138 (18.1%)
Missing	11 (1.5%)	4 (0.5%)
**Gestational age at birth (weeks)**		
Mean (SD)	38.8 (±2.2)	38.8 (±2.1)
Missing	1 (0.1%)	1 (0.1%)
**Smoking in pregnancy**		
Non-smoker	510 (70.6%)	576 (75.6%)
Smoker	153 (21.2%)	140 (18.4%)
Missing	59 (8.2%)	46 (6.0%)
**Any maternal psychiatric illness**		
Absent	697 (96.5%)	738 (96.9%)
present	14 (1.9%)	20 (2.6%)
Missing	11 (1.5%)	4 (0.5%)
**Maternal alcohol consumption in first three months**		
Any alcohol	342 (47.4%)	388 (50.9%)
Never	369 (51.1%)	370 (48.6%)
Missing	11 (1.5%)	4 (0.5%)

†Family income was recorded in 1990, explaining the low absolute monetary level.

Supplementary Table 3. Analytic vs excluded cohort

Demographics for analytic cohort vs excluded Raine Study participants
(total n = 2868)

	Excluded cohort (n = 1384)	Analytic cohort (n = 1484)
**Maternal age at birth (years)**		
Mean (SD)	27.2 (±6.0)	28.9 (±5.8)
Missing	57 (4.1%)	15 (1.0%)
**Female fetal sex**		
	692 (50.0%)	722 (48.7%)
**Income level†**		
Below 12.000 AUD	292 (21.1%)	179 (12.1%)
Above 12.000 AUD	937 (67.7%)	1,230 (82.9%)
Missing	155 (11.2%)	75 (5.1%)
**Maternal body mass index (kg/m^2)**		
Mean (SD)	22.2 (±4.4)	22.5 (±4.3)
Missing	50 (3.6%)	15 (1.0%)
**Maternal race**		
European descent	1,048 (75.7%)	1,424 (96.0%)
Other	287 (20.7%)	45 (3.0%)
Missing	49 (3.5%)	15 (1.0%)
**Maternal level of education**		
< 12 years	747 (54.0%)	701 (47.2%)
> 12 years	588 (42.5%)	768 (51.8%)
Missing	49 (3.5%)	15 (1.0%)
**Diabetes or hypertension in pregnancy**		
absent	1,126 (81.4%)	1,205 (81.2%)
present	209 (15.1%)	264 (17.8%)
Missing	49 (3.5%)	15 (1.0%)
**Gestational age at birth (weeks)**		
Mean (SD)	38.5 (±2.6)	38.8 (±2.2)
Missing	9 (0.7%)	2 (0.1%)
**Smoking in pregnancy**		
Non-smoker	819 (59.2%)	1,086 (73.2%)
Smoker	351 (25.4%)	293 (19.7%)
Missing	214 (15.5%)	105 (7.1%)
**Any maternal psychiatric illness**		
Absent	1,303 (94.1%)	1,435 (96.7%)
present	32 (2.3%)	34 (2.3%)
Missing	49 (3.5%)	15 (1.0%)
**Maternal alcohol consumption in first three months**		
Any alcohol	549 (39.7%)	730 (49.2%)
Never	786 (56.8%)	739 (49.8%)
Missing	49 (3.5%)	15 (1.0%)

†Family income was recorded in 1990, explaining the low absolute monetary level.

Supplementary Table 4. Measured birthweight

	Aggression problems		Attention problems		Social problems	
	**Model 1**	**Model 2**	**Model 1**	**Model 2**	**Model 1**	**Model 2**
**Main effect (normalized birth weight (SD))**	B: -0.0618<br>*CI: [-0.313, 0.191]<br>SE: 0.105<br>*P-value: 0.558	NA	B: -0.0954<br>*CI: [-0.216, 0.0256]<br>SE: 0.0505<br>*P-value: 0.059	NA	B: -0.0604<br>*CI:[-0.135, 0.014]<br>SE: 0.0312<br>*P-value: 0.0532	NA
**Female effect**	NA	B: -0.0460941<br>*CI: [-0.370, 0.286]<br>SE: 0.1372888<br>*P-value: 0.737	NA	B: -0.0273968<br>*CI: [-0.174, 0.111]<br>SE: 0.05864<br>*P-value: 0.64	NA	B: -0.0046495<br>*CI: [-0.113, 0.101]<br>SE: 0.04478380<br>*P-value: 0.917
**Male effect**	NA	B: -0.198<br>*CI: [-0.601, 0.199]<br>SE: 0.168<br>*P-value: 0.238	NA	B: -0.250<br>*CI:[-0.43, -0.0696]<br>SE: 0.0757<br>*P-value: 0.00096	NA	B: -0.128<br>*CI:[-0.232, -0.024]<br>SE: 0.0436<br>*P-value: 0.0033
**Interaction on female effect from male sex**	NA	B: -0.1519129<br>*CI: [-0.673, 0.345]<br>SE: 0.2133396<br>*P-value: 0.476	NA	B: -0.2224331<br>*CI: [-0.454, 0.003]<br>SE: 0.0955<br>*P-value: 0.0199	NA	B: -0.1235780<br>*CI: [-0.271, 0.027]<br>SE: 0.06245463<br>*P-value: 0.0479

Supplementary Table 4: Regressing the measured birthweight on scores of aggression, attention and social problems in males and females together and with the inclusion of an age interaction the regression output for our linear mixed effects model.

* p-value was calculated based on the z-statistic from the bootstrapped standard error, and the CI represents the 98.3% confidence interval.

Supplementary Table 5. Aggression with age two assessment

	**Model 1**	**Model 2**
**Main effect**	B: 0.141<br>*CI: [-0.119, 0.391]<br>SE: 0.104<br>*P-value: 0.187476	NA
**Female effect**	NA	B: 0.511<br>*CI: [0.164, 0.849]<br>SE: 0.14342248<br>*P-value: 0.00037
**Male interaction**	NA	B: -0.734<br>*CI: [-1.25, -0.237]<br>SE: 0.213<br>*P-value: 0.000561

Supplementary Table 5: Sensitivity analysis with inclusion of the age two preschool assessment showing the regression output for our linear mixed effects model.

* p-value was calculated based on the z-statistic from the bootstrapped standard error, and the CI represents the 98.3% confidence interval.

Supplementary Table 6. Term born only

	Aggression Problems	Attention Problems	Social Problems
**Female effect**	B: 0.387<br>*CI: [0.011, 0.762]<br>SE: 0.157<br>*P-value: 0.014	B: 0.142<br>*CI: [-0.0318, 0.311]<br>SE: 0.072<br>*P-value: 0.048	B: 0.0521<br>*CI: [-0.085, 0.182]<br>SE: 0.056<br>*P-value: 0.35151
**Male interaction**	B: -0.646<br>*CI: [-1.19, -0.114]<br>SE: 0.226<br>*P-value: 0.0042	B: -0.148<br>*CI: [-0.389, 0.0982]<br>SE: 0.102<br>*P-value: 0.148	B: -0.0158<br>*CI: [-0.186, 0.152]<br>SE: 0.071<br>*P-value: 0.823

Supplementary Table 6: Sensitivity analysis with term-born only showing the regression output for our linear mixed effects model.

* p-value was calculated based on the z-statistic from the bootstrapped standard error, and the CI represents the 98.3% confidence interval.

Supplementary Table 7. Teacher assessments

	Aggression Problems	Attention Problems	Social Problems
**Female effect**	B: 0.113<br>*CI: [-0.349, 0.573]<br>SE: 0.193<br>*P-value: 0.559	B: 0.138<br>*CI: [-0.432, 0.7]<br>SE: 0.237<br>*P-value: 0.561	B: -0.029<br>*CI: [-0.32, 0.263]<br>SE: 0.122<br>*P-value: 0.813
**Male interaction**	B: -0.792<br>*CI: [-1.625, 0.079]<br>SE: 0.357<br>*P-value: 0.026	B: -0.785<br>*CI: [-1.705, 0.177]<br>SE: 0.394<br>*P-value: 0.0463	B: -0.15<br>*CI: [-0.538, 0.239]<br>SE: 0.163<br>*P-value: 0.357

Supplementary Table 7: supplementary analysis using teacher assessments at age ten showing the regression output for our linear model after bootstrapping standard errors and confidence intervals.

* p-value was calculated based on the z-statistic from the bootstrapped standard error, and the CI represents the 98.3% confidence interval.

Supplementary Table 8. Sex interactions when using BW-PGS2

	Attention Problems	Aggression Problems	Social Problems
**Female effect**	B: 0.125<br>*CI: [-0.0394, 0.288]<br>SE: 0.069<br>*P-value: 0.068	B: 0.292<br>*CI: [-0.0618, 0.647]<br>SE: 0.148<br>*P-value: 0.049	B: 0.0552<br>*CI: [-0.0574, 0.167]<br>SE: 0.047<br>*P-value: 0.24
**Male interaction**	B: -0.139<br>*CI: [-0.370, 0.101]<br>SE: 0.099<br>*P-value: 0.160	B: -0.510<br>*CI: [-1.01, -0.00477]<br>SE: 0.210<br>*P-value: 0.0149	B: -0.0496<br>*CI: [-0.195, 0.100]<br>SE: 0.062<br>*P-value: 0.422

Supplementary Table 8: Sensitivity analysis using an older polygenic score with regression output for our linear mixed effects model. **BW-PGS2** = Secondary BW polygenic score made from an older GWAS.

* p-value was calculated based on the z-statistic from the bootstrapped standard error, and the CI represents the 98.3% confidence interval.

Supplementary Table 9. Including principal components

	Attention Problems	Aggression Problems	Social Problems
**Female effect**	B: 0.181<br>*CI: [0.0213, 0.335]<br>SE: 0.0657<br>*P-value: 0.006	B: 0.415<br>*CI: [0.0711, 0.747]<br>SE: 0.1417<br>*P-value: 0.003	B: 0.0756<br>*CI: [-0.0458, 0.194]<br>SE: 0.0502<br>*P-value: 0.1318
**Male interaction**	B: -0.190<br>*CI: [-0.421, 0.0411]<br>SE: 0.0968<br>*P-value: 0.049	B: -0.5887<br>*CI: [-1.08, -0.0804]<br>SE: 0.2096<br>*P-value: 0.005	B: -0.0420<br>*CI: [-0.202, 0.116]<br>SE: 0.0666<br>*P-value: 0.528

Supplementary Table 9: Sensitivity analysis including principal components to investigate potential confounding from population stratification. Adjusted for sex, age, and principal components with clustering at the participant level

* p-value was calculated based on the z-statistic from the bootstrapped standard error, and the CI represents the 98.3% confidence interval.

Supplementary Table 10. Temporal stability of sex differences

Interaction term	Attention Problems	Aggression Problems	Social Problems
**BW-PGS * sex**	B: -0.259<br>*CI: [-0.695, 0.187]<br>SE: 0.1845<br>*P-value: 0.16	B: -0.948<br>*CI: [-1.87, -0.0128]<br>SE: 0.3890<br>*P-value: 0.014771	B: -0.108<br>*CI: [-0.373, 0.173]<br>SE: 0.114<br>*P-value: 0.346
**Age * BW-PGS * Sex**	B: 0.00652<br>*CI: [-0.0267, 0.0394]<br>SE: 0.0139<br>*P-value: 0.638	B: 0.0340<br>*CI: [-0.0338, 0.102]<br>SE: 0.0284<br>*P-value: 0.230378	B: 0.00618<br>*CI: [-0.0153, 0.0264]<br>SE: 0.0087<br>*P-value: 0.48

Supplementary Table 10: Sensitivity analysis with inclusion of a three-way interaction (Age x Sex x BW-PGS) to test the stability of the sex differences across childhood and adolescence.

* p-value was calculated based on the z-statistic from the bootstrapped standard error, and the CI represents the 98.3% confidence interval.
